# Synthesis and crystal structure of 4,6-di­amino-1-cyclo­hexyl-1,3,5-triazine-2(1*H*)-thione monohydrate

**DOI:** 10.1107/S2056989026002240

**Published:** 2026-03-05

**Authors:** Ebtsam A. Ahmed, Benson M. Kariuki, Reham A. Mohamed-Ezzat, Rasha A. Azzam, Galal H. Elgemeie

**Affiliations:** a Department of Chemistry, Faculty of Science, Capital University, Helwan, Egypt; b Department of Chemistry College of Science King Faisal University, 31982 Al-Ahsa Saudi Arabia; c School of Chemistry, Cardiff University, Main Building, Park Place, Cardiff CF10, 3AT, United Kingdom; d Chemistry of Natural & Microbial Products Department, Pharmaceutical and Drug Industries Research Institute, National Research Centre, Cairo, Egypt; Tokyo University of Science, Japan

**Keywords:** synthesis, crystal structure, triazine­thione

## Abstract

In the crystal structure of the title compound, C_9_H_15_N_5_S·H_2_O, the di­amino­triazine­thione (DTT) moiety and water mol­ecules are hydrogen bonded to form ribbons. In the ribbon, each water mol­ecule accepts a pair of N—H⋯O bonds from the DTT moieties of two adjacent mol­ecules and donates one O—H⋯N bond to a third DTT moiety. The ribbons are stacked and linked through O—H⋯N inter­actions. The cyclo­hexane moieties are pendant to the ribbons forming a layer-like structure.

## Abstract 2

In the crystal structure of the title compound, the di­amino­triazine­thione (DTT) moiety and water mol­ecules are hydrogen bonded to form ribbons.

## 1. Chemical context

Triazines constitute one of the most noteworthy heterocyclic scaffolds for drug discovery as a result of their structural significance and broad-spectrum biological potencies (Kciuk *et al.*, 2023 ▸; Gornowicz *et al.*, 2020 ▸; Mohamed-Ezzat & Elgemeie, 2024*a* ▸; Abdallah *et al.*, 2021 ▸). Numerous triazine-containing drugs for treatment of many diseases have been approved by the FDA, including decitabine, cyclo­guanil, altretamine, bimiralisib, almitrine, lamotrigine and tri­aza­virin (Ali *et al.*, 2025 ▸).

The discovery of sulfur-based therapies has also been an important development of the pharmaceutical industry and sulfur-derived functional groups are present in a wide variety of natural products and pharmaceuticals. Sulfur continues to be the predominant heteroatom in many anti­metabolic heterocycles and in a range of FDA-approved drugs (Mohamed-Ezzat *et al.*, 2022 ▸, 2023 ▸; Feng *et al.*, 2016 ▸; Elgemeie *et al.*, 1992 ▸, 1999 ▸).

The results presented here were obtained in continuation of our program in the synthesis of heterocycles utilizing cyano­carboimidodi­thio­ate as a key precursor. This highly reactive compound has been utilized effectively in the synthesis of various heterocycles (Elgemeie & Mohamed, 2014*a* ▸,*b* ▸; Elgemeie *et al.*, 2015 ▸; Mohamed-Ezzat & Elgemeie, 2023 ▸, 2024*a* ▸; Mohamed-Ezzat *et al.*, 2024 ▸). We have also recently synthesized numerous triazines via novel approaches (Mohamed-Ezzat *et al.*, 2024*b* ▸, 2025 ▸).

**Figure 1:**
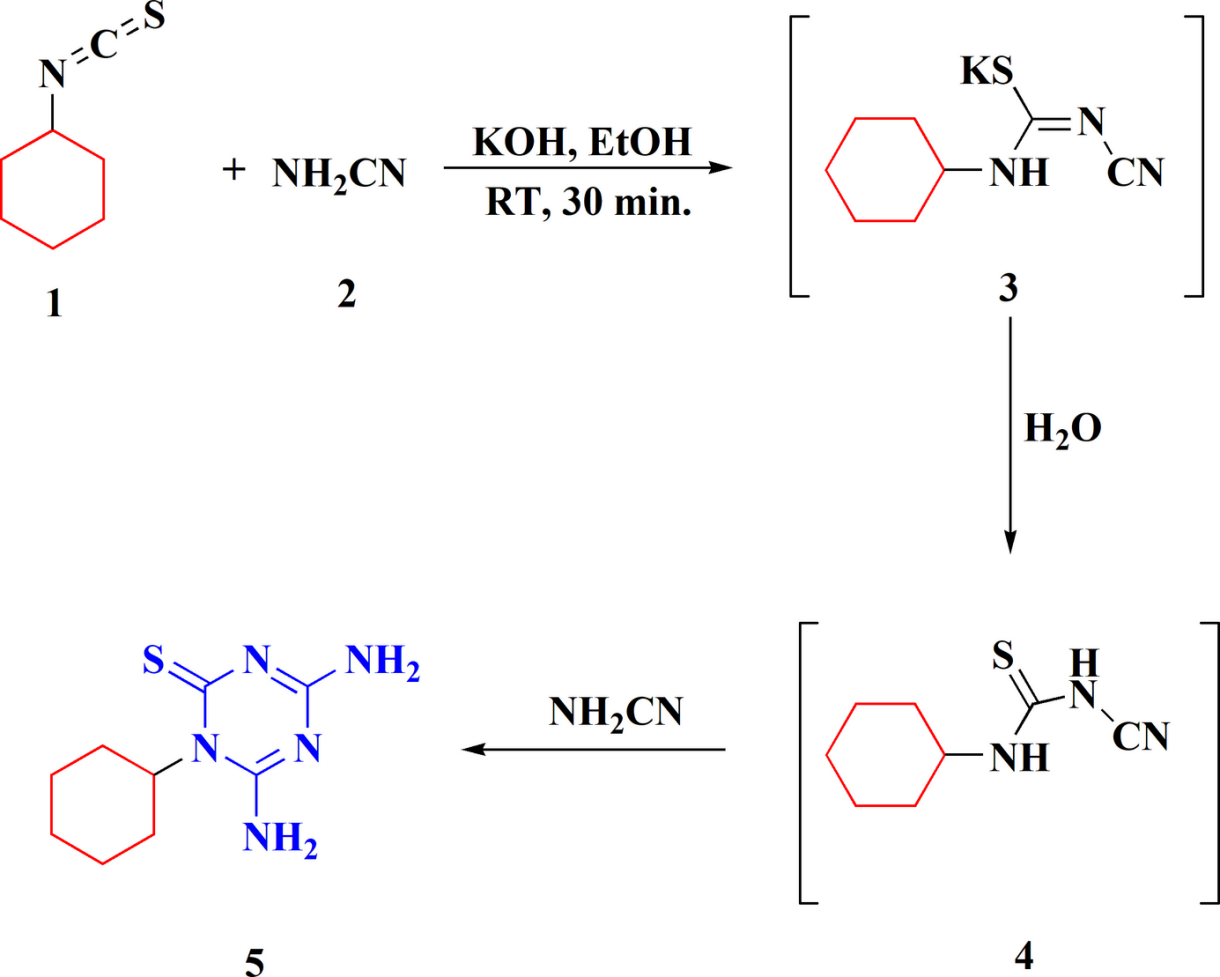
Reaction scheme showing the synthesis of compound **5**.

The incorporation of sulfur functionalities into mol­ecules containing the triazine ring system combines two privileged scaffolds. Combination of the two important pharmacophores in the framework is a strategy for the development of potentially novel therapeutic agents. Herein, the novel triazine­thione was synthesized via reaction of cyclo­hexylisothio­cyanate with cyanamide in the presence of potassium hydroxide at room temperature as depicted in Fig. 1 ▸.
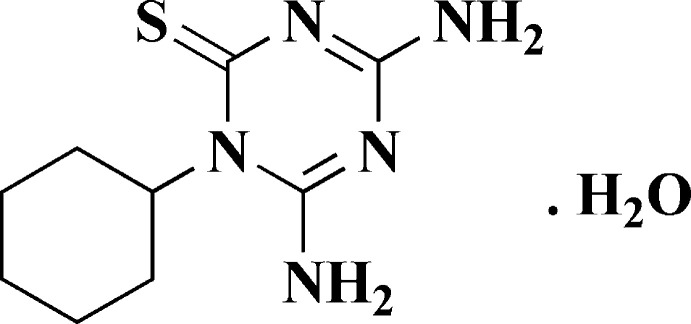


## 2. Structural commentary

**Figure 2:**
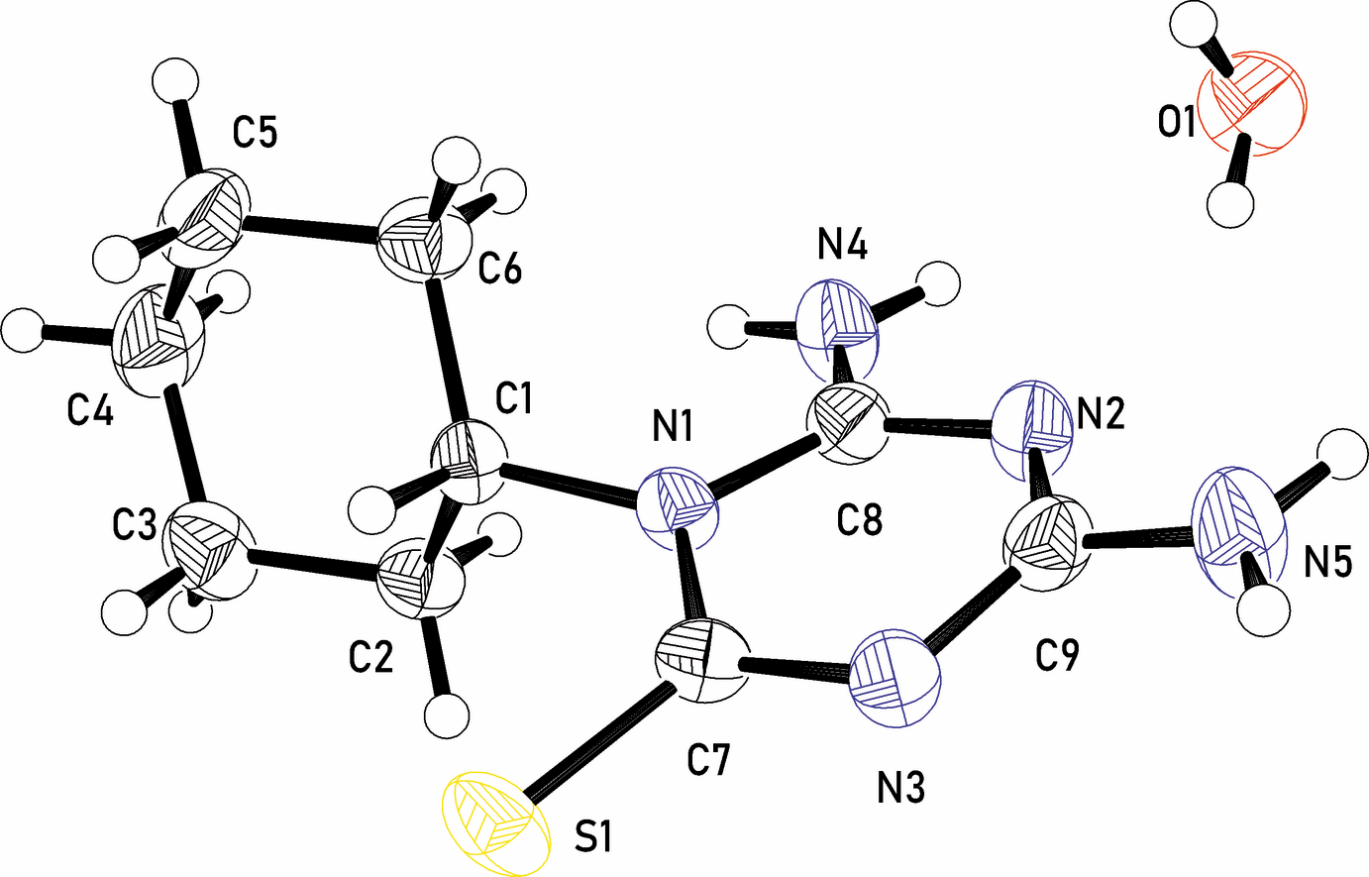
The asymmetric unit of the crystal structure of **5**·H_2_O showing displacement ellipsoids at the 50% probability level.

The compound crystallizes in the ortho­rhom­bic, *Pbca* space group. The asymmetric unit contains a mol­ecule of 4,6-di­amino-1-cyclo­hexyl-1,3,5-triazine-2(1*H*)-thione (**5**) and a water mol­ecule (Fig. 2 ▸). The mol­ecule of **5** consists of a cyclo­hexane ring (C1–C6) and a di­amino­triazine­thione (DTT) moiety (C7–C9, N1–N5, S1). In the mol­ecule, the least-squares plane through the cyclo­hexane ring is twisted from the plane through the DTT moiety by a dihedral angle of 72.07 (10)°.

The cyclo­hexane ring is in a chair conformation. The triazine ring of the DTT moiety is slightly curved as indicated by displacement of atoms C9 and N1 to the same side of the ring, away from the plane through atoms C7, C8, N2, N3, by 0.125 (3) and 0.161 (3) Å, respectively.

## 3. Supra­molecular features

**Figure 3:**
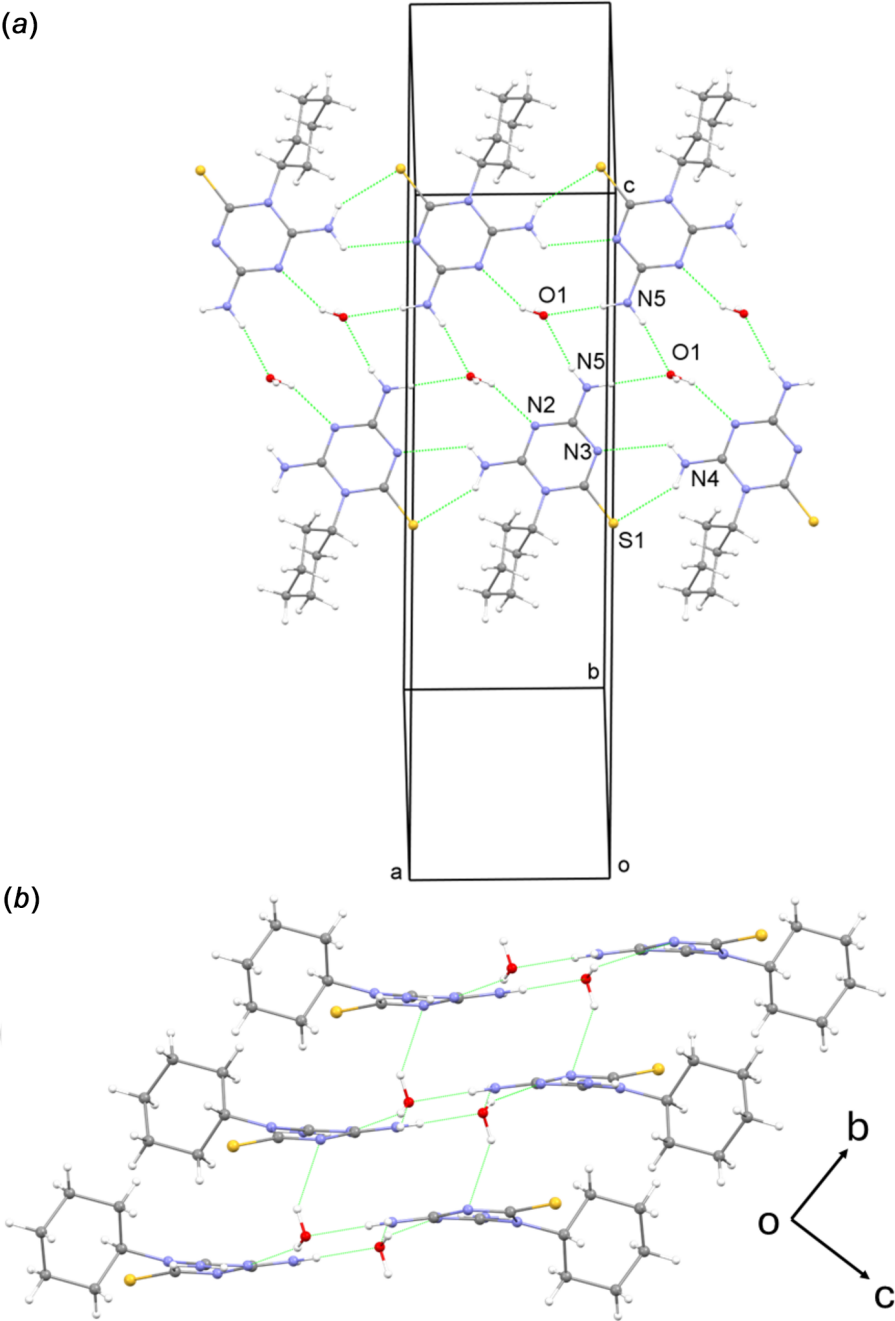
Segments of the crystal structure of **5**·H_2_O showing (*a*) a ribbon formed through hydrogen bonding and (*b*) the stacking of ribbons viewed down the *a* axis.

In the crystal structure, the DTT moieties and water mol­ecules are hydrogen bonded to form ribbons propagated in the [100] direction (Table 1 ▸, Fig. 3 ▸*a*). In the ribbon, each water mol­ecule accepts a pair of N—H⋯O bonds from the DTT moieties of two adjacent mol­ecules and donates one O—H⋯N bond to a third DTT moiety. Additional N—H⋯N and N—H⋯S hydrogen bonds also occur in the ribbon. The ribbons are stacked in the [010] direction in the crystal and they are linked through O—H⋯N inter­actions (Fig. 3 ▸*b*). Additionally, a weak O-H⋯S hydrogen bond connects ribbons in the *b-*axis direction. The cyclo­hexane moieties are pendant to the ribbons and hence the structure is layer-like.

## 4. Database survey

A search of the CSD (version 6.00, November 2025; Groom *et al.*, 2016 ▸) for crystal structures containing the di­amino­triazine­thione moiety revealed 6-(benzyl­sulfan­yl)-1,3,5-triazine-2,4-di­amine (COFPEW; Liu *et al.*, 2024 ▸) and bis­(4,6-di­amino-2-thiono-1*H*-(1,3,5)triazinium) aqua­bis­(oxalato-*O*,*O*′)dioxouranium(VI) *N*-cyano­guanidine (QELQAA, QELQAA01); Serezhkina *et al.*, 2007 ▸). COFPEW has three molecules in the asymmetric unit with the methylbenzene moieties linked to the DTT through the S atoms. The planes through the phenyl rings are twisted from the DTT planes by dihedral angles in the range 70–83°, comparable to that observed for the cyclohexane ring in the title compound. QELQAA(01) is a metal complex containing separate DTT units.

## 5. Synthesis and crystallization

The title compound was obtained, as depicted in Fig. 1 ▸, starting from the reaction of cyclo­hexyl­iso­thiocyanate (**1**) with cyanamide (**2**) in the presence of potassium hydroxide in ethanol at room temperature for 30 min. The reaction mixture was then poured into water and hydrolysed using hydro­chloric acid to give the title compound **5** as colorless crystals of various shapes which were then crystallized from water to give needles.

## 6. Refinement

Crystal data, data collection and structure refinement details are summarized in Table 2 ▸. The cyclo­hexane H atoms were inserted in idealized positions and refined using a riding model with *U*_iso_(H) = *U*_eq_(C).

## Supplementary Material

Crystal structure: contains datablock(s) I. DOI: 10.1107/S2056989026002240/jp2026sup1.cif

Structure factors: contains datablock(s) I. DOI: 10.1107/S2056989026002240/jp2026Isup3.hkl

Supporting information file. DOI: 10.1107/S2056989026002240/jp2026Isup3.cml

CCDC reference: 2531186

Additional supporting information:  crystallographic information; 3D view; checkCIF report

## Figures and Tables

**Table 1:** Hydrogen-bond geometry (Å, °)

*D*—H⋯*A*	*D*—H	H⋯*A*	*D*⋯*A*	*D*—H⋯*A*
N4—H4*C*⋯N3^i^	0.86 (3)	2.62 (3)	3.151 (3)	121 (2)
N4—H4*C*⋯O1	0.86 (3)	2.53 (3)	3.239 (3)	140 (2)
N4—H4*D*⋯S1^i^	0.87 (3)	2.52 (3)	3.313 (2)	152 (2)
N5—H5*C*⋯O1^ii^	0.89 (4)	2.09 (4)	2.967 (3)	167 (3)
N5—H5*D*⋯O1^iii^	0.83 (3)	2.29 (3)	3.094 (3)	164 (3)
O1—H1*O*⋯N2	0.82 (4)	2.19 (3)	2.949 (3)	153 (3)
O1—H2*O*⋯N3^iv^	0.85 (3)	2.61 (3)	3.325 (3)	142 (3)
O1—H2*O*⋯S1^iv^	0.85 (3)	2.79 (3)	3.574 (2)	153 (3)

Symmetry codes: (i) 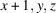; (ii) 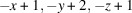; (iii) 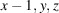; (iv) 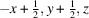.

**Table 2:** Experimental details

Crystal data	
Chemical formula	C_9_H_15_N_5_S·H_2_O
*M*_r_	243.33
Crystal system, space group	Orthorhombic, *Pbca*
Temperature (K)	293
*a*, *b*, *c* (Å)	7.0790 (4), 9.9029 (6), 33.0489 (19)
*V* (Å^3^)	2316.8 (2)
*Z*	8
Radiation type	Mo *K*α
μ (mm^−1^)	0.27
Crystal size (mm)	0.48 × 0.18 × 0.07

Data collection	
Diffractometer	SuperNova, Dual, Cu at home/near, Atlas
Absorption correction	Gaussian (*CrysAlis PRO*; Rigaku, 2024 ▸)
*T*_min_, *T*_max_	0.545, 1.000
No. of measured, independent and observed [*I* > 2σ(*I*)] reflections	20982, 3038, 2038
*R*_int_	0.076
(sin θ/λ)_max_ (Å^−1^)	0.697

Refinement	
*R*[*F*^2^ > 2σ(*F*^2^)], *wR*(*F*^2^), *S*	0.056, 0.136, 1.09
No. of reflections	3038
No. of parameters	167
H-atom treatment	H atoms treated by a mixture of independent and constrained refinement
Δρ_max_, Δρ_min_ (e Å^−3^)	0.35, −0.23

Computer programs: *CrysAlis PRO* (Rigaku, 2024 ▸), *SHELXT* (Sheldrick, 2015*a* ▸), *SHELXL* (Sheldrick, 2015*b* ▸), *ORTEP-3 for Windows* and *WinGX* (Farrugia, 2012 ▸).

## supplementary crystallographic information

### 4,6-Diamino-1-cyclohexyl-1,3,5-triazine-2(1H)-thione monohydrate Crystal data

**Table:**

C_9_H_15_N_5_S·H_2_O	*D*_x_ = 1.395 Mg m^−^^3^
*M**_r_* = 243.33	Mo *K*α radiation, λ = 0.71073 Å
Orthorhombic, *P**b**c**a*	Cell parameters from 5364 reflections
*a* = 7.0790 (4) Å	θ = 3.7–28.9°
*b* = 9.9029 (6) Å	µ = 0.27 mm^−^^1^
*c* = 33.0489 (19) Å	*T* = 293 K
*V* = 2316.8 (2) Å^3^	Needle, colourless
*Z* = 8	0.48 × 0.18 × 0.07 mm
*F*(000) = 1040	

### 4,6-Diamino-1-cyclohexyl-1,3,5-triazine-2(1H)-thione monohydrate Data collection

**Table:**

SuperNova, Dual, Cu at home/near, Atlas diffractometer	2038 reflections with *I* > 2σ(*I*)
Detector resolution: 10.5082 pixels mm^-1^	*R*_int_ = 0.076
ω scans	θ_max_ = 29.7°, θ_min_ = 3.1°
Absorption correction: gaussian (CrysAlisPro; Rigaku, 2024)	*h* = −9→8
*T*_min_ = 0.545, *T*_max_ = 1.000	*k* = −12→13
20982 measured reflections	*l* = −43→45
3038 independent reflections	

### 4,6-Diamino-1-cyclohexyl-1,3,5-triazine-2(1H)-thione monohydrate Refinement

**Table:**

Refinement on *F*^2^	0 restraints
Least-squares matrix: full	Hydrogen site location: mixed
*R*[*F*^2^ > 2σ(*F*^2^)] = 0.056	H atoms treated by a mixture of independent and constrained refinement
*wR*(*F*^2^) = 0.136	*w* = 1/[σ^2^(*F*_o_^2^) + (0.0441*P*)^2^ + 1.2175*P*] where *P* = (*F*_o_^2^ + 2*F*_c_^2^)/3
*S* = 1.09	(Δ/σ)_max_ < 0.001
3038 reflections	Δρ_max_ = 0.35 e Å^−^^3^
167 parameters	Δρ_min_ = −0.23 e Å^−^^3^

### 4,6-Diamino-1-cyclohexyl-1,3,5-triazine-2(1H)-thione monohydrate Special details

**Table:**

Geometry. All esds (except the esd in the dihedral angle between two l.s. planes) are estimated using the full covariance matrix. The cell esds are taken into account individually in the estimation of esds in distances, angles and torsion angles; correlations between esds in cell parameters are only used when they are defined by crystal symmetry. An approximate (isotropic) treatment of cell esds is used for estimating esds involving l.s. planes.

### 4,6-Diamino-1-cyclohexyl-1,3,5-triazine-2(1H)-thione monohydrate Fractional atomic coordinates and isotropic or equivalent isotropic displacement parameters (Å^2^)

**Table:**

	*x*	*y*	*z*	*U*_iso_*/*U*_eq_	
C1	0.3805 (3)	0.5848 (2)	0.34849 (6)	0.0293 (5)	
H1	0.265632	0.575172	0.332190	0.035*	
C2	0.4606 (3)	0.4428 (2)	0.35160 (7)	0.0353 (5)	
H2A	0.373765	0.385546	0.366454	0.042*	
H2B	0.579721	0.444867	0.366096	0.042*	
C3	0.4910 (4)	0.3861 (3)	0.30934 (8)	0.0466 (6)	
H3A	0.552642	0.298912	0.311366	0.056*	
H3B	0.369353	0.372666	0.296438	0.056*	
C4	0.6104 (4)	0.4792 (3)	0.28334 (8)	0.0537 (7)	
H4A	0.737921	0.482095	0.294053	0.064*	
H4B	0.616642	0.443384	0.256053	0.064*	
C5	0.5306 (4)	0.6210 (3)	0.28206 (7)	0.0470 (6)	
H5A	0.409229	0.619649	0.268428	0.056*	
H5B	0.614785	0.678590	0.266612	0.056*	
C6	0.5059 (3)	0.6795 (2)	0.32441 (7)	0.0378 (5)	
H6A	0.627880	0.688390	0.337523	0.045*	
H6B	0.448385	0.768242	0.322833	0.045*	
C7	0.1215 (3)	0.6284 (2)	0.39842 (6)	0.0314 (5)	
C8	0.4316 (3)	0.6997 (2)	0.41602 (6)	0.0320 (5)	
C9	0.1762 (3)	0.7788 (2)	0.44935 (6)	0.0355 (5)	
N1	0.3141 (2)	0.63940 (18)	0.38824 (5)	0.0297 (4)	
N2	0.3644 (3)	0.7730 (2)	0.44638 (5)	0.0371 (5)	
N3	0.0539 (3)	0.6996 (2)	0.42924 (6)	0.0364 (5)	
N4	0.6155 (3)	0.6863 (2)	0.41318 (7)	0.0416 (5)	
N5	0.1052 (4)	0.8655 (3)	0.47590 (7)	0.0531 (6)	
S1	−0.02187 (9)	0.52678 (7)	0.37155 (2)	0.0437 (2)	
H4C	0.687 (4)	0.731 (3)	0.4295 (7)	0.045 (7)*	
H4D	0.678 (4)	0.637 (3)	0.3961 (8)	0.052 (8)*	
H5C	0.185 (5)	0.915 (3)	0.4903 (10)	0.076 (10)*	
H5D	−0.011 (4)	0.874 (3)	0.4776 (8)	0.046 (8)*	
O1	0.6834 (3)	0.9457 (2)	0.47110 (6)	0.0525 (5)	
H1O	0.582 (5)	0.905 (3)	0.4719 (9)	0.063*	
H2O	0.654 (5)	0.993 (3)	0.4505 (9)	0.063*	

### 4,6-Diamino-1-cyclohexyl-1,3,5-triazine-2(1H)-thione monohydrate Atomic displacement parameters (Å^2^)

**Table:**

	*U*^11^	*U*^22^	*U*^33^	*U*^12^	*U*^13^	*U*^23^
C1	0.0252 (12)	0.0335 (11)	0.0292 (10)	0.0015 (9)	0.0002 (9)	−0.0035 (8)
C2	0.0342 (13)	0.0314 (11)	0.0401 (13)	0.0003 (9)	0.0014 (10)	0.0002 (9)
C3	0.0475 (16)	0.0380 (13)	0.0544 (15)	0.0028 (12)	0.0037 (12)	−0.0124 (11)
C4	0.0534 (18)	0.0636 (18)	0.0442 (14)	0.0065 (14)	0.0130 (13)	−0.0130 (13)
C5	0.0467 (16)	0.0609 (17)	0.0334 (12)	−0.0022 (13)	0.0064 (11)	0.0032 (11)
C6	0.0360 (14)	0.0351 (12)	0.0423 (13)	0.0000 (10)	0.0045 (10)	0.0012 (10)
C7	0.0254 (12)	0.0305 (11)	0.0382 (12)	0.0011 (9)	0.0004 (9)	0.0015 (9)
C8	0.0290 (12)	0.0343 (12)	0.0327 (11)	−0.0013 (9)	−0.0012 (9)	0.0003 (9)
C9	0.0302 (13)	0.0441 (13)	0.0321 (11)	0.0000 (10)	0.0042 (9)	−0.0020 (10)
N1	0.0207 (9)	0.0341 (10)	0.0341 (9)	−0.0015 (8)	−0.0004 (7)	−0.0044 (7)
N2	0.0288 (11)	0.0491 (12)	0.0332 (10)	−0.0004 (9)	−0.0001 (8)	−0.0080 (9)
N3	0.0257 (10)	0.0453 (11)	0.0382 (10)	−0.0032 (8)	0.0042 (8)	−0.0079 (9)
N4	0.0243 (11)	0.0550 (14)	0.0455 (12)	0.0014 (10)	−0.0041 (9)	−0.0145 (10)
N5	0.0295 (14)	0.0732 (17)	0.0567 (14)	−0.0022 (12)	0.0078 (11)	−0.0297 (12)
S1	0.0250 (3)	0.0452 (4)	0.0609 (4)	−0.0053 (3)	0.0014 (3)	−0.0173 (3)
O1	0.0395 (12)	0.0657 (14)	0.0522 (11)	−0.0120 (9)	−0.0087 (9)	0.0006 (9)

### 4,6-Diamino-1-cyclohexyl-1,3,5-triazine-2(1H)-thione monohydrate Geometric parameters (Å, º)

**Table:**

C1—N1	1.496 (3)	C6—H6B	0.9700
C1—C6	1.517 (3)	C7—N3	1.328 (3)
C1—C2	1.519 (3)	C7—N1	1.408 (3)
C1—H1	0.9800	C7—S1	1.683 (2)
C2—C3	1.520 (3)	C8—N4	1.312 (3)
C2—H2A	0.9700	C8—N2	1.326 (3)
C2—H2B	0.9700	C8—N1	1.375 (3)
C3—C4	1.517 (4)	C9—N5	1.327 (3)
C3—H3A	0.9700	C9—N2	1.337 (3)
C3—H3B	0.9700	C9—N3	1.344 (3)
C4—C5	1.515 (4)	N4—H4C	0.86 (3)
C4—H4A	0.9700	N4—H4D	0.87 (3)
C4—H4B	0.9700	N5—H5C	0.89 (4)
C5—C6	1.525 (3)	N5—H5D	0.83 (3)
C5—H5A	0.9700	O1—H1O	0.82 (4)
C5—H5B	0.9700	O1—H2O	0.85 (3)
C6—H6A	0.9700		
			
N1—C1—C6	114.88 (17)	H5A—C5—H5B	107.9
N1—C1—C2	113.10 (17)	C1—C6—C5	108.28 (19)
C6—C1—C2	112.89 (18)	C1—C6—H6A	110.0
N1—C1—H1	104.9	C5—C6—H6A	110.0
C6—C1—H1	104.9	C1—C6—H6B	110.0
C2—C1—H1	104.9	C5—C6—H6B	110.0
C1—C2—C3	109.42 (19)	H6A—C6—H6B	108.4
C1—C2—H2A	109.8	N3—C7—N1	119.42 (19)
C3—C2—H2A	109.8	N3—C7—S1	120.31 (17)
C1—C2—H2B	109.8	N1—C7—S1	120.28 (16)
C3—C2—H2B	109.8	N4—C8—N2	117.7 (2)
H2A—C2—H2B	108.2	N4—C8—N1	120.5 (2)
C4—C3—C2	112.0 (2)	N2—C8—N1	121.7 (2)
C4—C3—H3A	109.2	N5—C9—N2	117.0 (2)
C2—C3—H3A	109.2	N5—C9—N3	117.4 (2)
C4—C3—H3B	109.2	N2—C9—N3	125.5 (2)
C2—C3—H3B	109.2	C8—N1—C7	117.35 (18)
H3A—C3—H3B	107.9	C8—N1—C1	123.59 (17)
C5—C4—C3	111.8 (2)	C7—N1—C1	119.05 (17)
C5—C4—H4A	109.3	C8—N2—C9	115.88 (19)
C3—C4—H4A	109.3	C7—N3—C9	117.17 (19)
C5—C4—H4B	109.3	C8—N4—H4C	119.0 (17)
C3—C4—H4B	109.3	C8—N4—H4D	127.5 (19)
H4A—C4—H4B	107.9	H4C—N4—H4D	114 (3)
C4—C5—C6	111.7 (2)	C9—N5—H5C	118 (2)
C4—C5—H5A	109.3	C9—N5—H5D	118.9 (19)
C6—C5—H5A	109.3	H5C—N5—H5D	123 (3)
C4—C5—H5B	109.3	H1O—O1—H2O	95 (3)
C6—C5—H5B	109.3		

### 4,6-Diamino-1-cyclohexyl-1,3,5-triazine-2(1H)-thione monohydrate Hydrogen-bond geometry (Å, º)

**Table:**

*D*—H···*A*	*D*—H	H···*A*	*D*···*A*	*D*—H···*A*
N4—H4*C*···N3^i^	0.86 (3)	2.62 (3)	3.151 (3)	121 (2)
N4—H4*C*···O1	0.86 (3)	2.53 (3)	3.239 (3)	140 (2)
N4—H4*D*···S1^i^	0.87 (3)	2.52 (3)	3.313 (2)	152 (2)
N5—H5*C*···O1^ii^	0.89 (4)	2.09 (4)	2.967 (3)	167 (3)
N5—H5*D*···O1^iii^	0.83 (3)	2.29 (3)	3.094 (3)	164 (3)
O1—H1*O*···N2	0.82 (4)	2.19 (3)	2.949 (3)	153 (3)
O1—H2*O*···N3^iv^	0.85 (3)	2.61 (3)	3.325 (3)	142 (3)
O1—H2*O*···S1^iv^	0.85 (3)	2.79 (3)	3.574 (2)	153 (3)

Symmetry codes: (i) *x*+1, *y*, *z*; (ii) −*x*+1, −*y*+2, −*z*+1; (iii) *x*−1, *y*, *z*; (iv) −*x*+1/2, *y*+1/2, *z*.
